# Supplementary material for: Identification of photoperiod-induced specific miRNAs in the adrenal glands of Sunite sheep (Ovis aries)
Source: Front Vet Sci. 2022 Jul 22;9:888207. doi: 10.3389/fvets.2022.888207 (PMC9354845; doi:10.3389/fvets.2022.888207)
Supplement: Supplementary file 1 [file Data_Sheet_1.ZIP › Supplementary file/miRDeep2 file.zip!/miRDeep2 file/Know_Structure/SPLP42A1/oar-mir-10a.pdf]

miRBase precursor : oar-mir-10a
Total read count : 76296
oar-miR-10a read count : 75800
remaining reads : 496

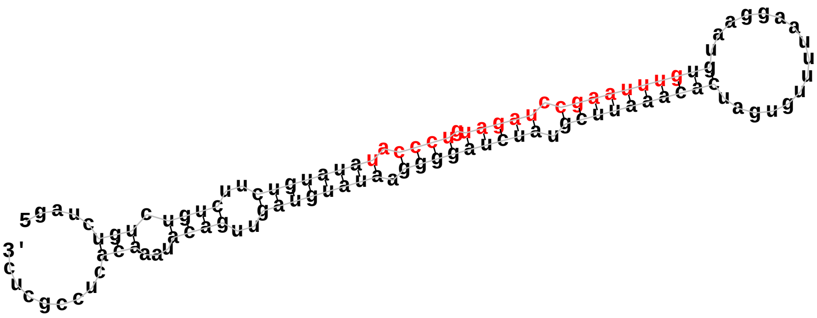

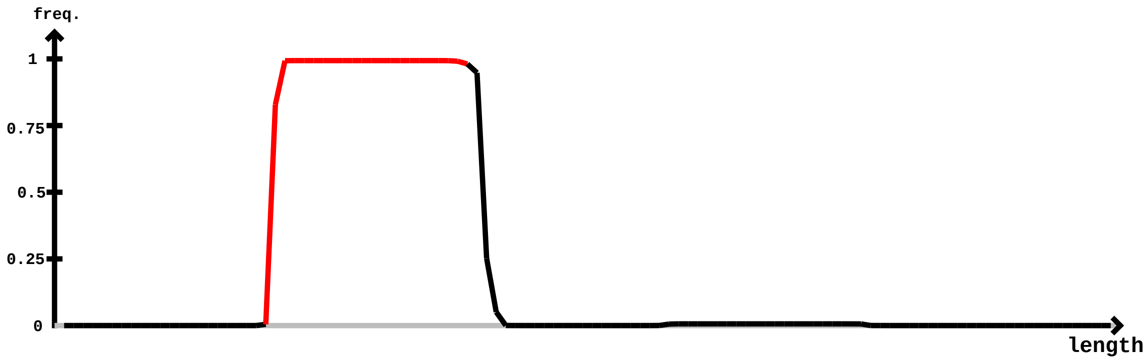


```
                                   oar-miR-10a
5'- gaucugucugucuucuguauauacccuguagauccgaauuuguguaaggaauuuugugaucacaaauucguaucuaggggaauauguaguugacauaaacacuccgcuc   -3'    exp
    ....(((.((((..((((((((.((((.(((((.((((((((((................)))))))))).))))))))).))))))))..))))...)))........    reads   mm   sample
    .aucugucugucuucuguaua........................................................................................    1       0    seq
    ..ucugucugucuucuguaua........................................................................................    1       0    seq
    ....................auacccuguagauccgaauu.....................................................................    2       0    seq
    ....................auacccuguagauccgaauuu....................................................................    19      0    seq
    ....................auacccuguagauccgaauuug...................................................................    49      0    seq
    ....................auacccuguagauccgaauuugu..................................................................    263     0    seq
    ....................auacccuguagauccgaauuugug.................................................................    21      0    seq
    ....................auacccuguagauccgaauuugugu................................................................    2       0    seq
    .....................uacccuguagauccgaau......................................................................    15      0    seq
    .....................uacccuguagauccgaauu.....................................................................    148     0    seq
    .....................uacccuguagauccgaauuu....................................................................    677     0    seq
    .....................uacccuguagauccgaauuug...................................................................    2153    0    seq
    .....................uacccuguagauccgaauuugu..................................................................    46796   0    seq
    .....................uacccuguagauccgaauuugug.................................................................    10867   0    seq
    .....................uacccuguagauccgaauuugugu................................................................    2169    0    seq
    .....................uacccuguagauccgaauuugugua...............................................................    10      0    seq
    .....................uacccuguagauccgaauuuguguaa..............................................................    1       0    seq
    .....................uacccuguagauccgaauuuguguaag.............................................................    1       0    seq
    ......................acccuguagauccgaau......................................................................    3       0    seq
    ......................acccuguagauccgaauu.....................................................................    19      0    seq
    ......................acccuguagauccgaauuu....................................................................    91      0    seq
    ......................acccuguagauccgaauuug...................................................................    311     0    seq
    ......................acccuguagauccgaauuugu..................................................................    5986    0    seq
    ......................acccuguagauccgaauuugug.................................................................    4485    0    seq
    ......................acccuguagauccgaauuugugu................................................................    1678    0    seq
    ......................acccuguagauccgaauuugugua...............................................................    6       0    seq
    ......................acccuguagauccgaauuuguguaa..............................................................    1       0    seq
    .......................cccuguagauccgaauuugu..................................................................    9       0    seq
    .......................cccuguagauccgaauuugug.................................................................    8       0    seq
    .......................cccuguagauccgaauuugugu................................................................    8       0    seq
    ........................ccuguagauccgaauuugug.................................................................    2       0    seq
    ........................ccuguagauccgaauuuguguaa..............................................................    1       0    seq
    ............................................uaaggaauuuugugauca...............................................    1       0    seq
    ............................................................cacaaauucguaucuaggggaau..........................    1       0    seq
    .............................................................acaaauucguaucuagggga............................    1       0    seq
```

oar-miR-10a

```
gaucugucugucuucuguauauacccuguagauccgaauuuguguaaggaauuuugugaucacaaauucguaucuaggggaauauguaguugacauaaacacuccgcuc
.............................................................acaaauucguaucuaggggaa...........................	2	0	seq
.............................................................acaaauucguaucuaggggaau..........................	19	0	seq
..............................................................caaauucguaucuagggg.............................	1	0	seq
..............................................................caaauucguaucuaggggа............................	9	0	seq
..............................................................caaauucguaucuaggggaa...........................	13	0	seq
..............................................................caaauucguaucuaggggaau..........................	373	0	seq
..............................................................caaauucguaucuaggggaaua.........................	5	0	seq
...............................................................aaauucguaucuaggggaau..........................	55	0	seq
...............................................................aaauucguaucuaggggaaua.........................	4	0	seq
...............................................................aaauucguaucuaggggaauau........................	5	0	seq
................................................................aauucguaucuaggggaauau........................	4	0	seq
```
